# *Clonorchis sinensis* excretory-secretory products increase malignant characteristics of cholangiocarcinoma cells in three-dimensional co-culture with biliary ductal plates

**DOI:** 10.1371/journal.ppat.1007818

**Published:** 2019-05-23

**Authors:** Jihee Won, Youngkyu Cho, Dahyun Lee, Bo Young Jeon, Jung-Won Ju, Seok Chung, Jhang Ho Pak

**Affiliations:** 1 School of Mechanical Engineering, Korea University, Seoul, Republic of Korea; 2 Department of IT Convergence, Korea University, Seoul, Republic of Korea; 3 Department of Convergence Medicine, University of Ulsan College of Medicine and Asan Institute for Life Sciences, Asan Medical Center, Seoul, Republic of Korea; 4 Division of Vectors & Parasitic Diseases, Korean Centers for Disease Control and Prevention, Osong, Republic of Korea; 5 KU-KIST Graduate School of Converging Science and Technology, Korea University, Seoul, Republic of Korea; George Washington University School of Medicine and Health Sciences, UNITED STATES

## Abstract

*Clonorchis sinensis* is a carcinogenic human liver fluke, prolonged infection which provokes chronic inflammation, epithelial hyperplasia, periductal fibrosis, and even cholangiocarcinoma (CCA). These effects are driven by direct physical damage caused by the worms, as well as chemical irritation from their excretory-secretory products (ESPs) in the bile duct and surrounding liver tissues. We investigated the *C*. *sinensis* ESP-mediated malignant features of CCA cells (HuCCT1) in a three-dimensional microfluidic culture model that mimics an *in vitro* tumor microenvironment. This system consisted of a type I collagen extracellular matrix, applied ESPs, GFP-labeled HuCCT1 cells and quiescent biliary ductal plates formed by normal cholangiocytes (H69 cells). HuCCT1 cells were attracted by a gradient of ESPs in a concentration-dependent manner and migrated in the direction of the ESPs. Meanwhile, single cell invasion by HuCCT1 cells increased independently of the direction of the ESP gradient. ESP treatment resulted in elevated secretion of interleukin-6 (IL-6) and transforming growth factor-beta1 (TGF-β1) by H69 cells and a cadherin switch (decrease in E-cadherin/increase in N-cadherin expression) in HuCCT1 cells, indicating an increase in epithelial-mesenchymal transition-like changes by HuCCT1 cells. Our findings suggest that *C*. *sinensis* ESPs promote the progression of CCA in a tumor microenvironment via the interaction between normal cholangiocytes and CCA cells. These observations broaden our understanding of the progression of CCA caused by liver fluke infection and suggest a new approach for the development of chemotherapeutic for this infectious cancer.

## Author summary

The oriental liver fluke, *Clonorchis sinensis*, is a biological carcinogen of humans and is the cause of death of infectious cancer patients in China and Korea. Its chronic infection promotes cholangiocarcinogenesis due to direct contact of host tissues with the worms and their excretory-secretory products (ESPs); however, the specific mechanisms underlying this pathology remain unclear. To assess its contribution to the progression of cholangiocarcinoma (CCA), we developed a 3-dimensional (3D) *in vitro* culture model that consists of CCA cells (HuCCT1) in direct contact with normal cholangiocytes (H69), which are subsequently exposed to *C*. *sinensis* ESPs; therefore, this model represents a *C*. *sinensis*-associated CCA microenvironment. Co-cultured HuCCT1 cells exhibited increased motility in response to *C*. *sinensis* ESPs, suggesting that this model may recapitulate some aspects of tumor microenvironment complexity. Proinflammatory cytokines such as IL-6 and TGF-β1 secreted by H69 cells exhibited a crosstalk effect regarding the epithelial-mesenchymal transition of HuCCT1 cells, thus, promoting an increase in the metastatic characteristics of CCA cells. Our findings enable an understanding of the mechanisms underlying the etiology of *C*. *sinensis*-associated CCA, and, therefore, this approach will contribute to the development of new strategies for the reduction of its high mortality rate.

## Introduction

Cholangiocarcinoma (CCA) is an aggressive malignancy of the bile duct epithelia associated with local invasiveness and a high rate of metastases. It is the second most common primary hepatic tumor after hepatocellular carcinoma, which is considered to be a highly lethal cancer with a poor prognosis due to the difficulty in accurate early diagnosis [1]. There are several established risk factors for CCA, including primary cholangitis, biliary cysts and hepatolithiasis [2]. Another critical factor is infection with the liver flukes *Opisthorchis viverrini* and *Clonorchis sinensis*, resulting in the highest incidences of CCA being in Southeast Asian countries [3].

The proposed mechanisms of liver fluke-associated cholangiocarcinogenesis include mechanical damage to bile duct epithelia resulting from the feeding activities of the worms, infection-related inflammation, and pathological effects from their excretory-secretory products (ESPs), consisting of a complex mixture of proteins and other metabolites) [4]. These coordinated actions provoke epithelial desquamation, adenomatous hyperplasia, goblet cell metaplasia, periductal fibrosis, and granuloma formation, all contributing to the production of a conducive tumor microenvironment. Eventually, malignant cholangiocytes undergo uncontrolled proliferation that leads to the initiation and progression of CCA [5].

Like other parasitic helminths, liver flukes release ESPs continuously during infection, in this case into bile ducts and surrounding liver tissues. These substances play pivotal roles in host–parasite interactions [6]. Exposure of human CCA cells and normal biliary epithelial cells to liver fluke ESPs results in diverse pathophysiological responses, including proliferation and inflammation [7, 8]. Additionally, profiling of differential cancer-related microRNAs (miRNAs) expression has revealed that the miRNAs involved in cell proliferation and the prevention of tumor suppression are dysregulated in both CCA cells and normal cholangiocytes exposed to *C*. *sinensis* ESPs [9]. These results suggest that there are ESP-responsive pathologic signal cascades that are common to both cancerous and non-cancerous bile duct epithelial cells.

Another aspect of carcinogenic transformation is the tissue microenvironment, consisting of the extracellular matrix (ECM) and surrounding cells and is a crucial factor in the regulation of cancer cell motility and malignancy [10]. The diverse responses of tumor cells, cholangiocytes, and immune cells in the CCA microenvironment cooperatively affect cancer progression, including invasion, and/or metastasis [11]. Chronic inflammation of the bile duct due to the presence of liver flukes is closely associated also with the development of CCA, because it causes biliary epithelial cells to produce various cytokines and growth factors including interleukin-6, -8 (IL-6, -8), transforming growth factor-β (TGF-β), tumor necrosis factor-α (TNF-α), platelet-derived growth factor and epithelial growth factor [12]. Exposure to cytokines and growth factors induces their endogenous production by CCA cells through a crosstalk loop, enhancing malignant features such as invasion, metastasis, chemoresistance and epithelial-mesenchymal transition (EMT) [13]. Cytokines driven by chronic inflammation contribute to the pathogenesis of CCA and should be collectively considered in studies on tumor microenvironment.

**Fig 1:**
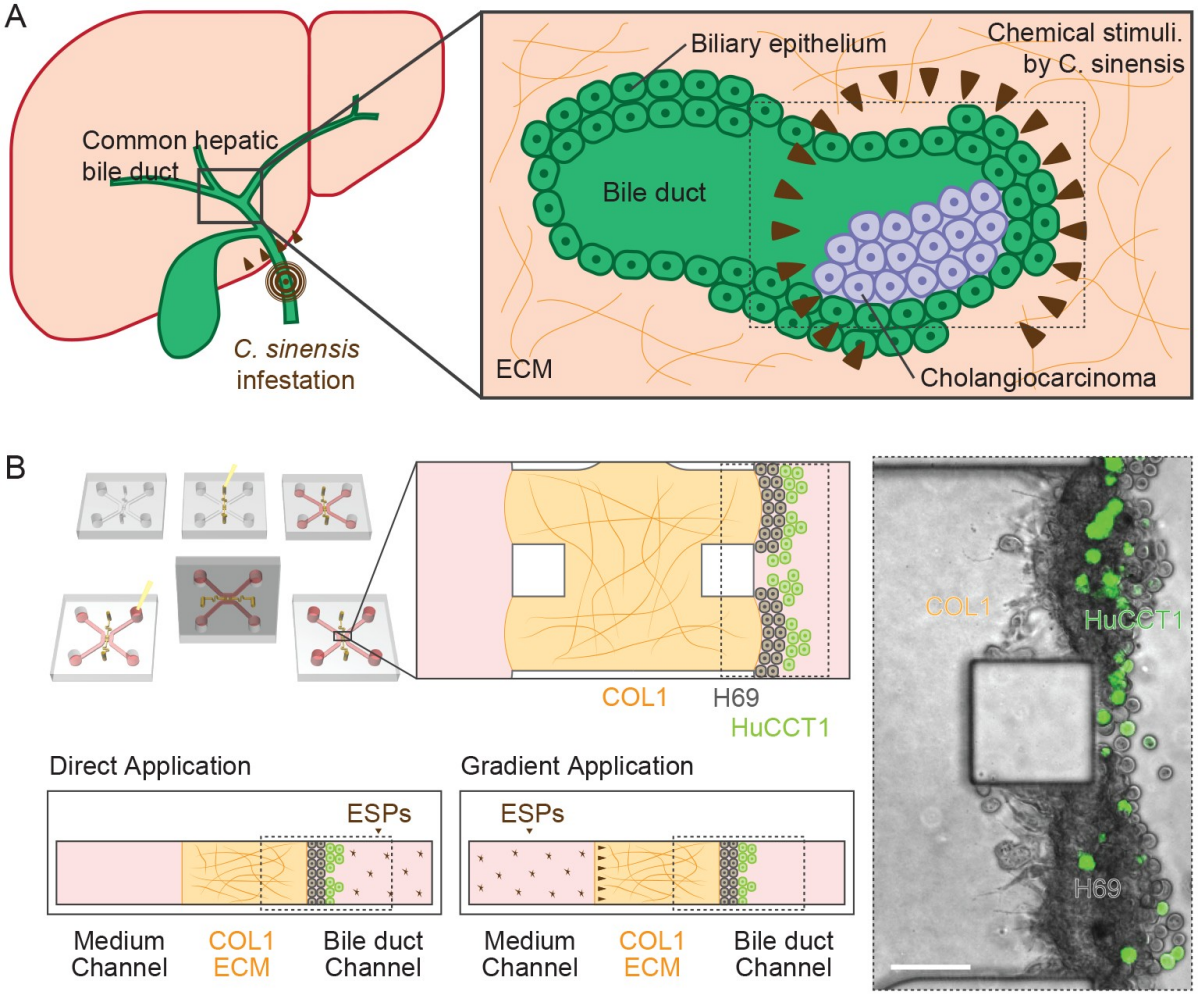
Physiological features of the human bile duct infected with *C*. *sinensis* and experimental design for assessing invasion by CCA cells in a clonorchiasis-associated tumor microenvironment. (A) A common hepatic bile duct cancer (hilar cholangiocarcinoma) and *C*. *sinensis* infestation of a human liver (left). Formation of a tumor gland in the bile duct and chemical stimulation by ESPs (brown triangles) from *C*. *sinensis* (right). (B) The schematic figure (box with black border) is a COL1 hydrogel scaffold region for cell culture. Phase-contrast image showing green fluorescence protein-expressing HuCCT1 CCA cells in a complex tumor microenvironment (box with dotted line in A and B) consisting of H69 normal cholangiocyte and the directional application of ESPs.

We have established a three-dimensional (3D) cell culture assay previously that contains a gradient of *C*. *sinensis* ESPs in the ECM and mimics the complex CCA microenvironment. In this previous study, CCA cells (HuCCT1) were morphologically altered to form aggregates in response to *C*. *sinensis* ESPs, and these CCA cells could only invade the type I collagen (COL1) hydrogel scaffold in response to ESP gradient treatment. This response was accompanied with an elevation of focal adhesion protein expression and the secretion of matrix metalloproteinase (MMP) isoforms [14], suggesting that *C*. *sinensis* ESPs may promote CCA progression. Additionally, this study revealed the chemoattractant effect of *C*. *sinensis* ESP gradients for CCA cells and to expand this work, we explored the more complicated tumor microenvironment subjected to ESPs from *C*. *sinensis*. In the present study, we developed an *in vitro* clonorchiasis-associated tumor microenvironment model that consisted of the following factors: (1) a 3D culture system of normal cholangiocytes using a microfluidic device as 3D quiescent biliary ductal plates on ECM; (2) physiological co-culture of CCA cells with normal cholangiocytes coupled to the directional application of *C*. *sinensis* ESPs to reconstitute a 3D CCA microenvironment; and (3) visualization and assessment of the interactions between tumor cells and their microenvironments to assess how the malignant progression of CCA corresponds with carcinogenic liver fluke infestation (Fig 1).

## Results

### Formation of *in vitro* 3D biliary ductal plate

**Fig 2:**
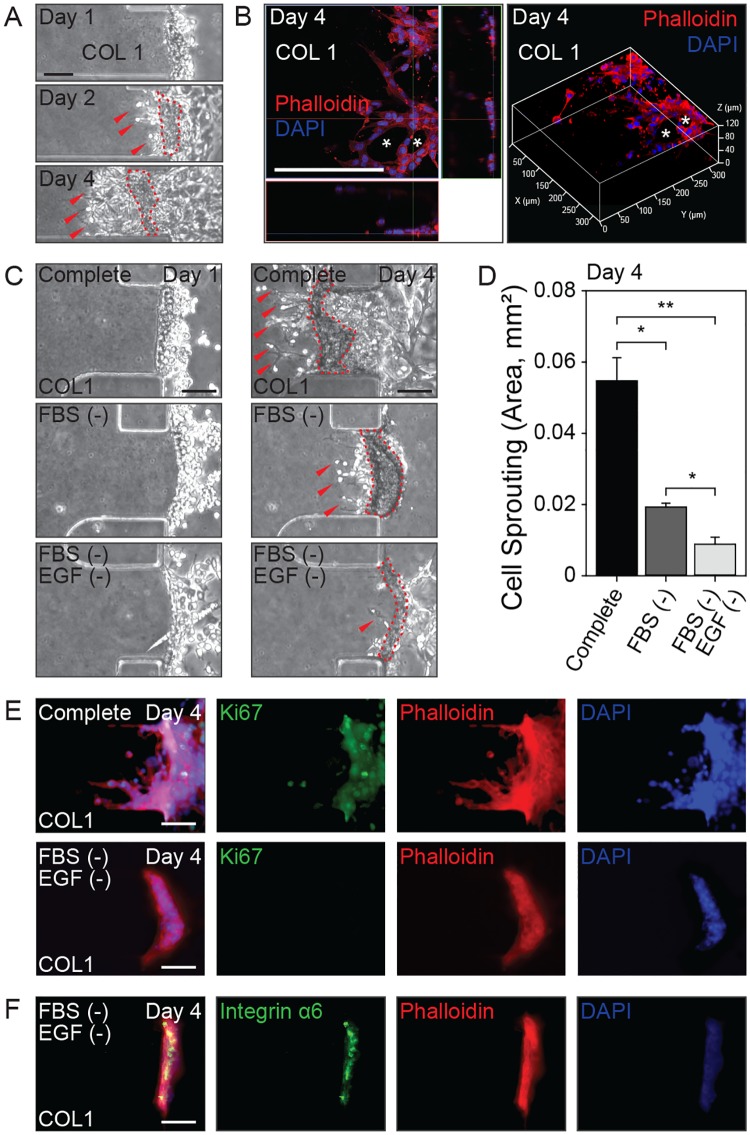
Features of H69 cells in 3D culture and development of a quiescent 3D biliary ductal plate. (A) Cholangiogenesis by H69 cells in 3D culture. Red dotted lines indicate initial formation of a ductal plate and arrows indicate 3-dimensionally sprouting primitive ductal structures. (B) An orthogonal confocal microscopic image of a mature duct structure enveloped by H69 cells sprouted into the COL1 scaffold (asterisks) visualized from above (x-y axis) in the center image and the z (height axis) in the right and below. A z-stack image shows the 3D structure of neoductule formation by sprouts of H69 cells. (C) H69 cells sprouting in media of differing compositions; complete (Complete), FBS-free (FBS (-)) and FBS-free/EGF-depleted (FBS (-)/EGF (-)). (D) Quantification of H69 cell sprouting into COL1 hydrogel (area). (E) Expression of Ki-67 (green) in H69 cells in complete (above) and FBS-free/EGF-depleted (below) media. (F) Expression and localization of integrin α6 (green) in H69 cells on the COL1 hydrogel scaffold. Scale bars = 100 μm (A, B, C, E and F). DAPI and phalloidin staining highlight nucleus in blue and actin cytoskeleton in red, respectively (B, E and F). *P < 0.05 and **P < 0.01 versus complete medium. Student’s *t*-test was used to analyze significance. Error bars = ± SEM.

To reconstitute the microenvironment of a normal bile duct on an ECM, H69 cells were cultured three dimensionally on a COL1 hydrogel within a microfluidic device. The cells formed an epithelial layer and sprouted 3-dimensionally into the hydrogel one day after seeding (Fig 2A). The sprouts formed 3D tube-like structures resembling newly-developed small bile ducts (Fig 2A and 2B). This morphological change can be referred to as cholangiogenesis, and hepatic neoductule formation from an existing biliary ductal plate [15].

The sprouting was suppressed in this study to form quiescent mature biliary ductal plates by varying the composition of the culture medium, namely complete, fetal bovine serum-free (FBS (-)), and FBS-free/epidermal growth factor-depleted (FBS (-)/EGF (-)). In complete culture medium, H69 cells dynamically sprouted and expanded into the COL1 hydrogel and the boundary between the biliary ductal plate and the COL1 hydrogel (Fig 2C, Day 4) moved far from the initial cell seeding point (Fig 2C, Day 1). In the absence of FBS and EGF, cholangiogenesis decreased dramatically (Fig 2D). Additionally, the H69 cells in FBS-free/EGF-depleted medium were in G_0_ phase (Fig 2E) and expressed a basolateral polarity marker (Integrin α6) along the region of the COL1 hydrogel scaffold that was in contact with the cell layer (Fig 2F) [16]. Therefore, we designated this cluster of H69 cells as representing a quiescent 3D biliary ductal plate.

**Fig 3:**
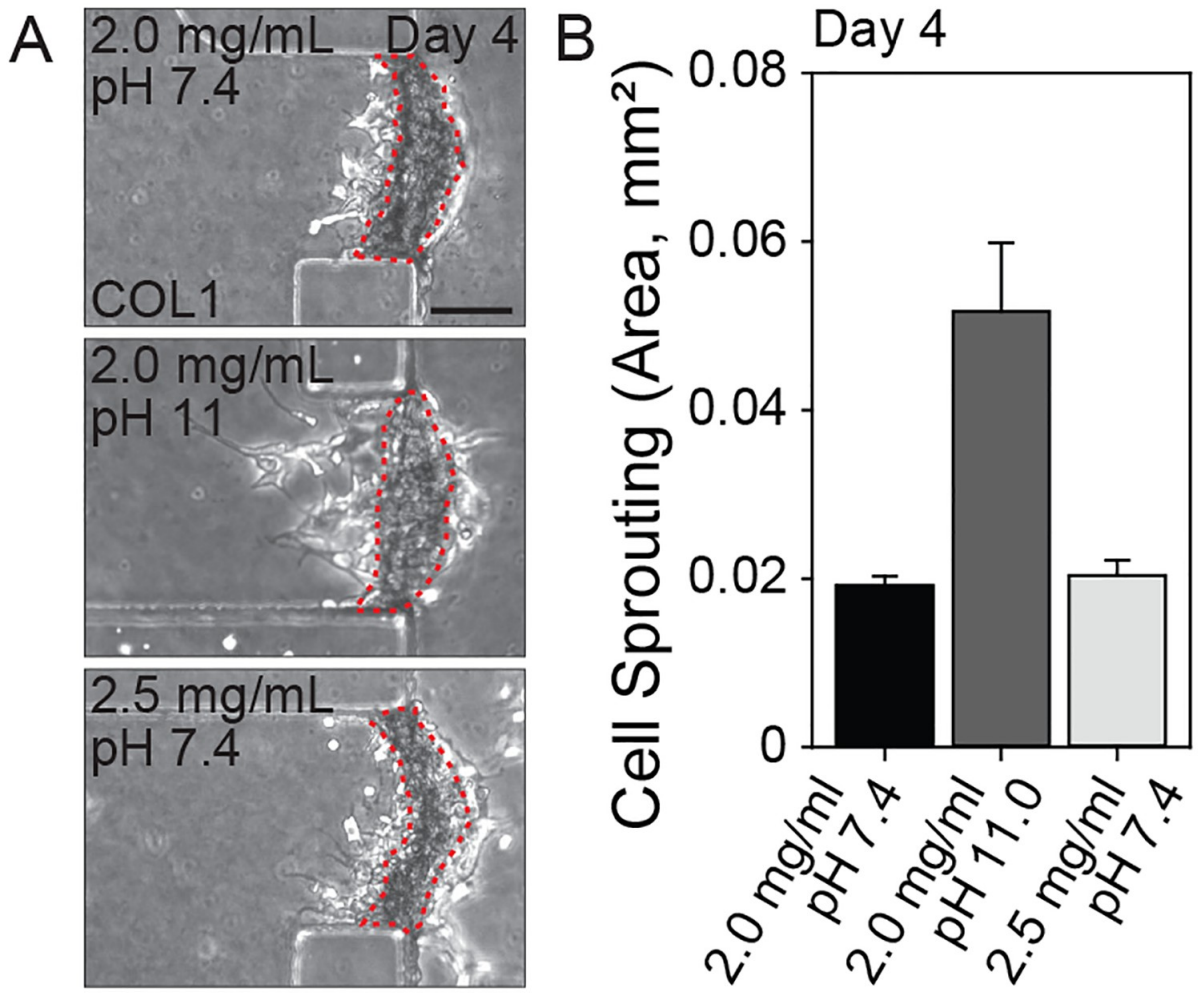
H69 cell sprouting into COL1 hydrogels with differing mechanical properties. (A) Sprouting of H69 cells under various ECM conditions; general control (2.0 mg/mL of COL1, pH 7.4), higher pH (2.0 mg/mL, pH 11), and higher concentration (2.5 mg/mL, pH 7.4). (B) Quantification of cell sprouting of H69 cells into COL1 hydrogels (area). Compared to the general control, there were no significant differences between groups. Scale bars = 100 μm (A). Student’s *t*-test was used to analyze significance. Error bars = ± SEM.

The mechanical properties of the COL1 hydrogel were modulated by altering the initial pH or concentration to identify other factors that could suppress the H69 cell sprouting. When the pH of the COL1 solution prior to gelation was basic (pH 11), the resulting hydrogel was stiffer than one gelled at pH 7.4 and one produced with a high concentration (2.5 mg/mL) [17]. H69 cells on stiffer COL1 hydrogel showed more numerous sprouts with larger surface areas (Fig 3A and 3B).

### Cholangiocarcinoma cell migration into 3D biliary ductal plates in the presence of ESPs

**Fig 4:**
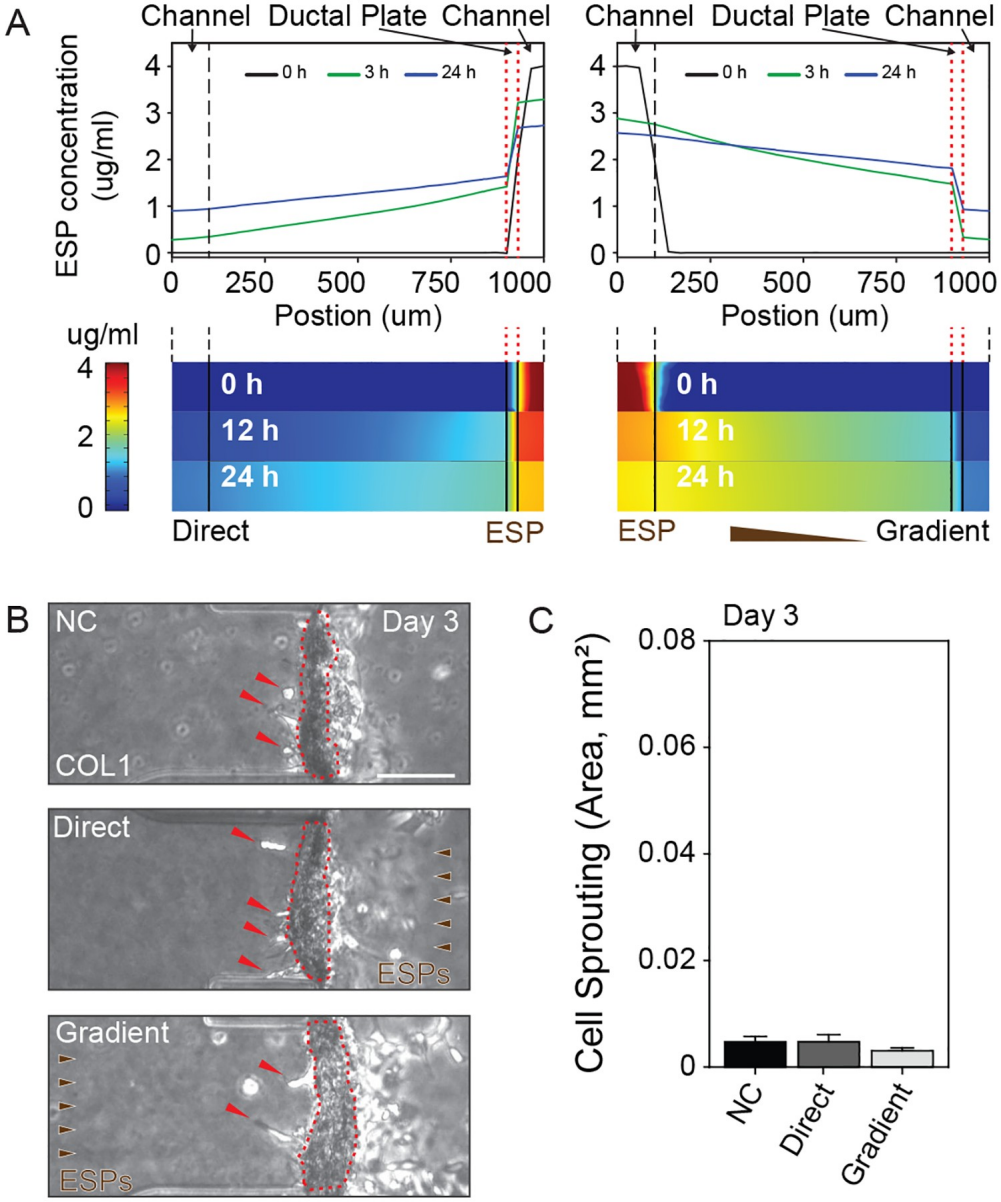
Behavioral changes in H69 cells in quiescent culture by the pathogenic effect of ESP treatment and the diffusion profile of ESPs in a COL1 hydrogel. (A) Our model predicts that ESP concentrations varied in the region of the biliary ductal plate (red dotted lines) between 1.5 μg/mL to 3.0 μg/mL and 1.7 μg/mL to 0.5 μg/mL after direct and gradient application respectively. Concentration profiles and heat-map images show the ESP concentration along the cell-seeded channel, COL1 hydrogel scaffold and opposite channel. (B) H69 cells sprouting induced by ESP treatment conditions; non-treated control (NC), direct treatment (Direct) and treatment with a gradient (Gradient). Dotted lines (red) and arrows (red) indicate the biliary ductal plate and H69 cell sprouting, respectively. (C) Quantification of the area of H69 cell sprouting into the COL1 hydrogel (area). There was no significant difference between the direct and gradient ESP applications and the NC. Scale bars = 100 μm (B). Student’s *t*-test was used to analyze significance. Error bars = ± SEM.

H69 cells cultured on normal COL1 hydrogels (2.0 mg/mL and pH 7.4) and in FBS (-)/EGF (-) medium formed a quiescent biliary ductal plate. To mimic *C*. *sinensis* infestation, ductal plates were treated with ESPs (4 μg/mL) by either application to the channel containing the H69 cells (direct application) or to the other channel of the microfluidic device (gradient application). After gradient application, ESPs diffused through the COL1 hydrogel and toward the basal side of the biliary duct plate, forming a complex concentration profile. Computational simulation results showed that after 24 hours ESP concentration reached 3 μg/mL at the apical side of the biliary ductal plate (2~2.5 μg/mL at basal) upon direct application, and 1.5~2 μg/mL at the basal side of the biliary ductal plate (1 μg/mL at apical) upon gradient application (Fig 4A). Based on the observation that the H69 cells produced three stratified layers and each layer is 10 μm in thick, ~40% of the local ESP concentration difference was applied to a single HuCCT1 cell entered the biliary ductal plate under gradient application. The 3D biliary ductal plate was stably maintained and remained healthy after either type of ESP treatment and neither treatment induced cholangiogenesis (Fig 4B and 4C).

**Fig 5:**
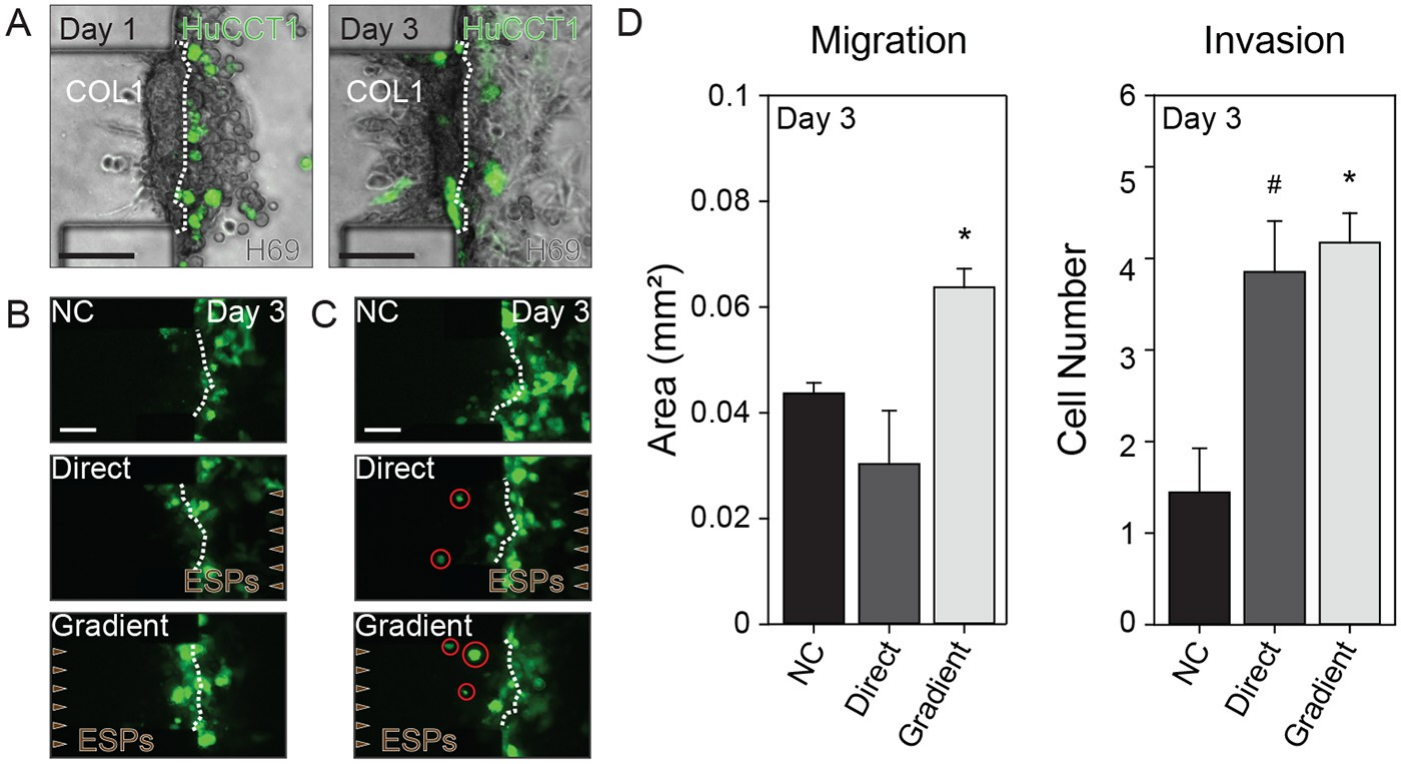
Increased migration and invasion of CCA cells after ESP treatment of the biliary tumor microenvironment. (A) H69 and HuCCT1 cells shown as a bright field image with green fluorescence overlay. Initial positions of H69 (unstained) and HuCCT1 cells (green) after serial cell seeding (left, Day 1) and subsequent invasion of HuCCT1 cells through the H69 cell layer in the microfluidic device (right, Day 3). (B) Migration of HuCCT1 cells toward the COL1 hydrogel scaffold on day 3. (C) Single cell invasion by HuCCT1 cells (red circle) into the COL1 hydrogel scaffold on day 3. (D) Quantified migration of HuCCT1 cells (penetrated area of the COL1 ECM) and invasion by HuCCT1 cells (counted number of individually invading cells). Green fluorescence indicates HuCCT1-GFP (A, B and C). Scale bars = 100 μm (A, B and C). #*P* = 0.058, *P < 0.05 versus non-treated control. Student’s *t*-test was used to analyze significance. Error bars = ± SEM.

HuCCT1 CCA cells labeled with GFP were seeded onto the apical side of the 3D quiescent biliary ductal plate formed by H69 cells under the culture condition as defined above. The HuCCT1 cells were then exposed to ESPs (direct or gradient) for 3 days. After the ESP treatment, the HuCCT1 cells actively invaded the biliary ductal plate and reached the COL1 hydrogel (Fig 5A). After gradient ESP application, 1.71-fold and 1.85-fold more HuCCT1 cells invaded the biliary ductal plate compared to non-treated control, or those treated with direct application, respectively (Fig 5B and 5D). Interestingly, the number of individualized HuCCT1 cells in the biliary duct layer and COL1 hydrogel were similar, both after gradient and direct treatment (Fig 5C and 5D).

### Elevated levels of proinflammatory cytokine secretion by H69 cells and EMT in HuCCT1 cells after ESP treatment

**Fig 6:**
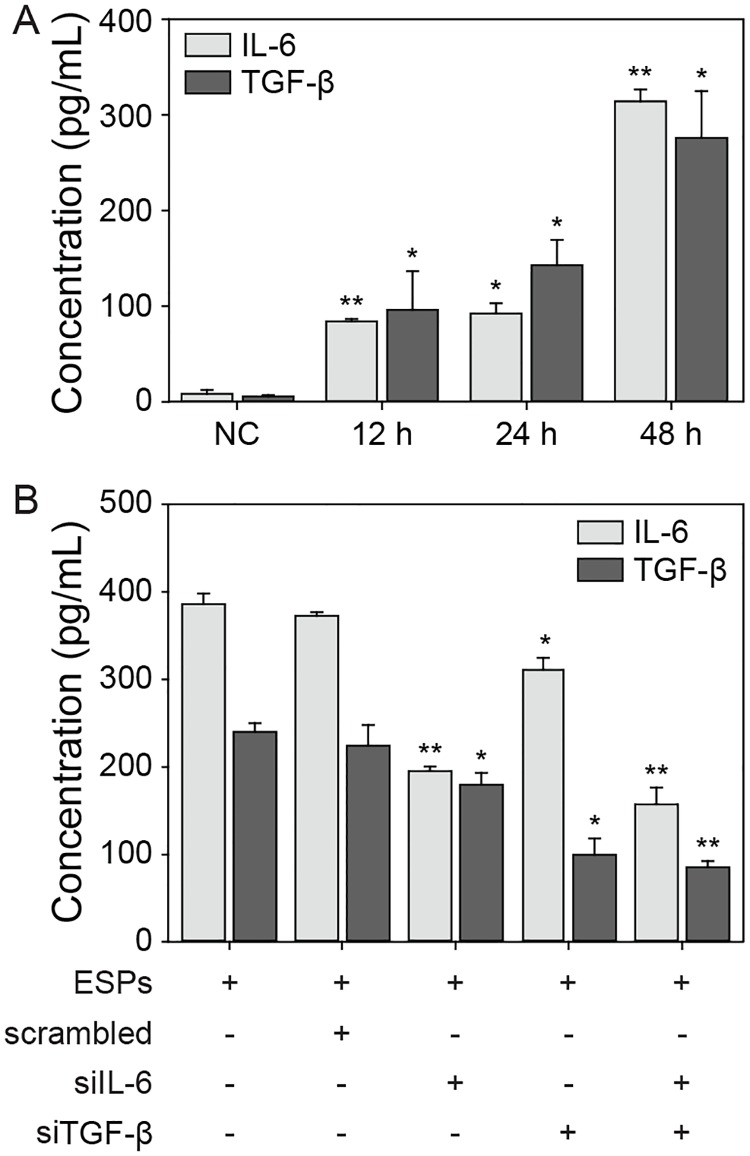
Expression of IL-6 and TGF-β1 in ESP-treated H69 and HuCCT cells. (A) H69 cells were treated with 800 ng/mL of ESPs for 0 (NC, non-treated control), 12, 24, and 48 hours, and the production of IL-6 or TGF-β1 in each culture supernatant was determined by ELISA. (B) HuCCT1cells were treated with 800 ng/mL of ESPs for 24 hours, and the medium was then replaced by culture supernatant from one of four groups of H69 siRNA transfectants exposed to ESPs for 48 hours. Cells were incubated further for 48 hours, and the production of IL-6 or TGF-β1 in each culture supernatant was determined by ELISA. The concentration of each cytokine was calculated from IL-6 and TGF-β1 standard curves. *P < 0.05, **P < 0.01 versus non-treated control. Student’s *t*-test was used to analyze significance. Error bars = ±SEM.

It has been reported that elevated plasma levels of IL-6 and TGF-β1 are correlated with histophathological changes in the livers of *C*. *sinensis*-infected mice [18, 19]. Moreover, interaction of these cytokines appears to be assoiciated with an increased malignancy of CCA cells [13]. These findings prompted us to examine whether IL-6 and TGF-β1 were involved in invasion and migration of HuCCT1 cells in our system. First, we measured IL-6 and TGF-β1 levels in the culture supernatants of ESP-treated H69 cells using ELISA, and found that secretion of both IL-6 and TGF-β1 was significantly elevated at 12 hours post-ESP treatment, compared to the non-treated control (Fig 6A). Elevated secretion of IL-6 was maintained at 24 hours and increased further increased by 48 hours, while the TGF-β1 secretion level increased in a time-dependent manner.

To assess the crosstalk of IL-6 and TGF-β1 from H69 cells with co-cultured HuCCT1 cells, the induction of IL-6 and TGF-β1 in ESP-treated H69 cells was attenuated by means of small interfering (si) RNA transfection. The culture supernatants from each of four groups of 48 hour-ESP-treated H69 cells (transfected with siRNAs of scrambled oligonucleotide or with siRNAs for IL-6, TGF-β1 or both) were substituted for 24 hour-ESP-treated medium in HuCCT1 cell cultures. Then, these HuCCT1 cells were incubated further for 48 hours and their culture supernatants were analyzed using ELISA. The levels of both ESP-induced IL-6 and TGF-β1 secretion by HuCCT1 cells, as well as H69 cells, were significantly decreased in the respective siRNA transfectants, when compared with those of untransfected and scrambled siRNA-transfected.cells (Fig 6B). Moreover, a greater reduction in the secretion of these cytokines was observed when using the supernatant from cells treated with siRNA for both IL-6 and TGF-β1 siRNA than when using ones from cells treated with either siRNA alone, suggesting that an IL-6/TGF-β1 autocrine/paracrine signaling network may be in effect between non-cancerous and cancerous co-cultured cells.

**Fig 7:**
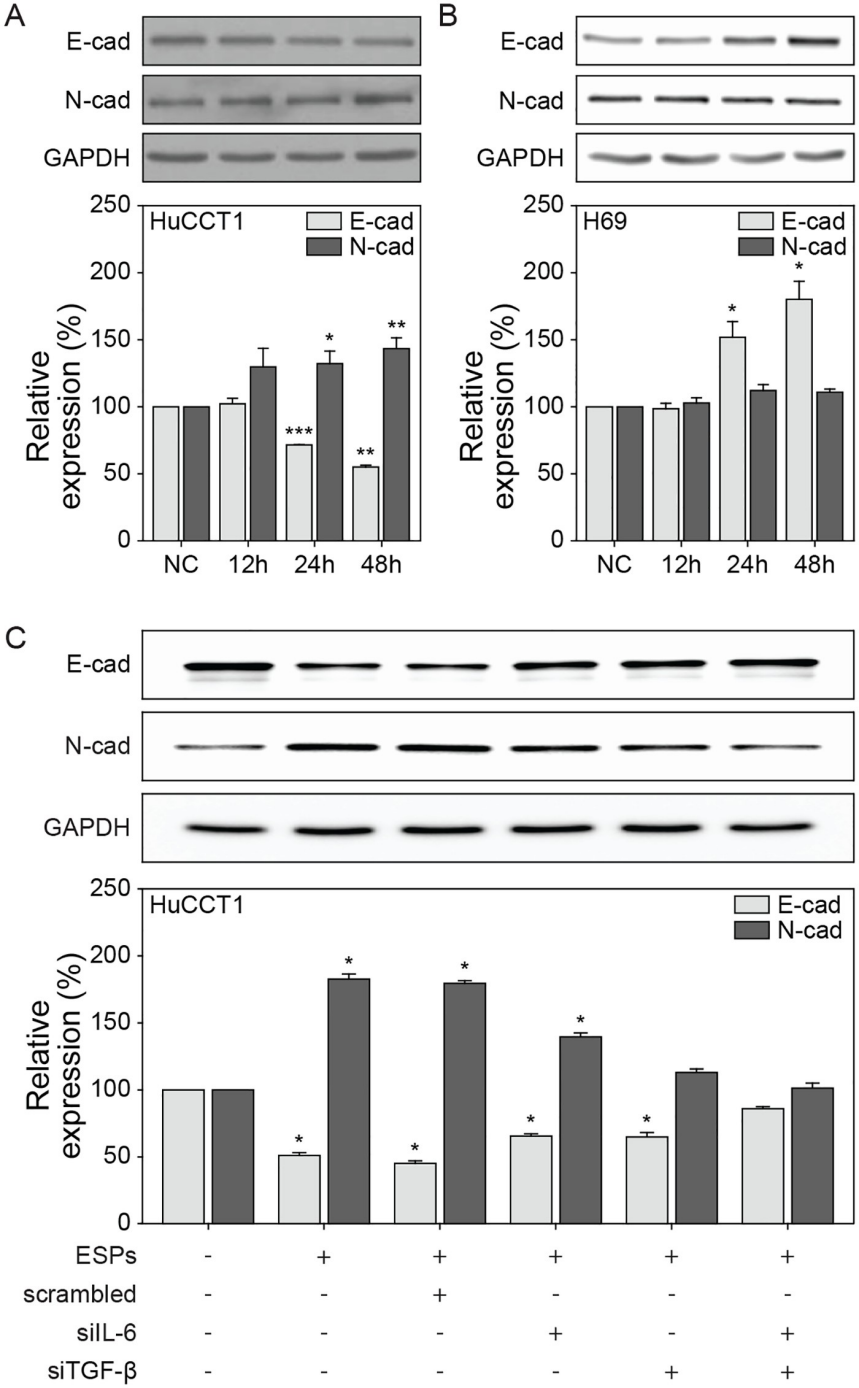
Expression of E- and N-cadherin in ESP-treated HuCCT1 and H69 cells. HuCCT1 (A) or H69 (B) cells were treated with 800 ng/mL of ESPs and harvested after 0–48 hours for immunoblot analysis of E- and N-cadherin expression. (C) The 24-hour ESP-treated medium of HuCCT1 cells was replaced by the culture supernatants from each four different H69 siRNA transfectants as described in the materials and methods section. After a 48-hour-incubation, total soluble proteins were immunoblotted using polyclonal antibodies against E- and N-cadherin. Protein bands were quantified densitometrically and normalized to the density of the GAPDH band. The ratio of E- or N-cadherin to GAPDH in each group is presented as its fold-change relative to the non-treated 0–hour control (NC). *P < 0.05, **P < 0.01 and ***P < 0.001 with control. Student’s *t*-test was used to analyze significance. Error bars = ± SEM.

Next, we examined ESP-mediated changes in E- and N-cadherin expression in HuCCT1 and H69 cells, which are, respectively, epithelial and mesenchymal markers, that are regarded as functionally significant factors in cancer progression. Decreased amounts of immunoreactive E-cadherin were detected in HuCCT1 cells following 24 hours post-ESP treatment, and this decreased further at 48 hours. Increased expression of N-cadherin was obvious at 24 hours post-ESP treatment and maintained up to 48 hours (Fig 7A). However, in H69 cells, the expression of E-cadherin was significantly elevated at 24 hours and increased further subsequently, while there was no substantial change in N-cadherin expression during the same period of ESP treatment (Fig 7B). This suggests that ESPs may contribute to facilitating EMT-like changes only in HuCCT1 cells, leading to the promotion of migration/invasion.

Finally, we evaluated the involvement of IL-6 and TGF-β1 in the cadherin switching of HuCCT1 cells treated with culture supernatants from siRNA-treated cells described above. Silencing of IL-6 and TGF-β1 markedly attenuated the reduction of E-cadherin and the elevation of N-cadherin expression induced by ESPs. The levels of E- and N-cadherin expression in double silencing supernatant-treated HuCCT1 cells were almost the same as those of the non-treated control (Fig 7C), indicating that the IL-6 and TGF-β1 expression induced by the ESPs contributed to EMT progression in HuCCT1 cells.

## Discussion

The microfluidic model of a CCA tumor microenvironment used in this study consisted of a quiescent 3D biliary ductal plate formed by H69 cells (cholangiocytes) on a COL1 ECM that has been stimulated by *C*. *sinensis* ESPs. HuCCT1 cells (CCA cells) responded to microenvironmental factors actively by proliferating, migrating and invading the 3D biliary ductal plate and passing into the neighboring ECM. HuCCT1 cells exhibited different cellular behaviors when co-cultured on the biliary duct layer, compared to when they were cultured on ECM alone, as described previously [14].

As a CCA tumor microenvironment factor, characteristics of normal cholangiocytes were carefully investigated, and compared with previous reports [20]. Ishida Y et al. reported ductular morphogenesis and functional polarization of human biliary epithelial cells when embedded three dimensionally in a COLI hydrogel [21]. Tanimizu N et al. also reported the development of a 3D tubular-like structure during the differentiation of mouse liver progenitor cells [16, 22]. However, these traditional dish-based culture platforms only generated 3D tube-like structures whose apical-basal polarities differed from those observed *in vivo*, and which were unsuitable for co-culturing with CCA cells to monitor tumor malignancy changes upon invasion/migration in a tumor milieu.

In the microfluidic 3D culture platform described here, H69 cells formed a cholangiocyte layer and sprouted into the COL1 hydrogel. This mimicked an asymmetrical ductal structure at the parenchymal layer on the portal vein side and a primitive ductal structure during the early stage of biliary tubulogenesis during cholangiogenesis [15]. H69 cholangiocytes lining small bile ducts are layer-forming biliary epithelial cells with a potential proliferative capacity, but under normal conditions are quiescent or in the G_0_ state of the cell cycle [23]. The mechanical and biochemical properties of the ECM and culture medium within the microenvironment of the biliary epithelium were characterized and shown to be conducive to the formation of a stable 3D biliary ductal plate and primitive ductal structure, which are crucial steps in cholangiogenesis.

The reconstituted biliary ductal plate on the ECM formed a 3D CCA tumor microenvironment, which was then seeded with CCA tumor cells and treated with *C*. *sinensis* ESPs. Many publications have described the co-culture of tumor cells with stromal cells (mainly fibroblasts) and reported upregulated tumor cell malignancy, however, only a few of these studied CCA cells [24]. One study co-cultured various CCA cells (HuCCT1 and MEC) with hepatic stellate cells as a CCA stroma and reported increased invasion and proliferation by CCA cells [25]. To our knowledge, this is the first attempt to construct a co-culture system that facilitates the direct contact of normal and CCA tumor cells from a single type of tissue; both cells were cultured separately and the combined to produce the pathophysiological effect. Therefore our work describes an advanced method for orchestrating complex CCA microenvironments, especially in 3D.

The first components of the CCA microenvironment are growth factors, which are present in the culture medium and are candidates for promoting the proliferation, differentiation and migration of cholangiocytes. FBS should be preferentially excluded to arrest the cells in a quiescent state in order to assess the direct effects of *C*. *sinensis* ESPs on CCA malignancy. Although the precise effect of EGF on normal cholangiocytes remains to be elucidated, key roles of EGF in biliary duct development and cholangiocytes differentiation including cholangiogenesis and neoductule formation from an existing biliary ductal plate, have been reported [16, 26]. Additionally, signaling via EGF and its receptor (EGFR) facilitates the progression of hepato-cholangiocellular cancer [27, 28]. Thus, we excluded EGF from our 3D co-culture model system because the complex and diverse roles of EGF might mask direct ESP-dependent effects on the CCA microenvironment.

The second component in the microenvironment was the COL1 ECM. The mechanical properties of the COL1 hydrogel, such as fibril diameter and stiffness, can be altered by controlling the collagen concentrations or adjusting the pH in of the collagen solution prior to gel casting. High pH reduces the diameter of COL1 nanofibers after gelation and this increases the stiffness of the COL1 hydrogel dominantly; the linear modulus of a COL1 hydrogel produced from pH 11 and 2.0 mg/mL is 2.7-fold and 3.1-fold higher than those from pH 7.4 and 2.5 mg/mL and from pH 7.4 and 2.0 mg/mL, being approximately 53 kPa, 20 kPa and 17 kPa, respectively [17]. It has been reported that hepatobiliary cells express diverse cellular behaviors with respect to proliferation, differentiation, adhesion, and migration under stiff ECM conditions, with close association in development, homeostasis and disease progression [29, 30]. The morphological changes in H69 cells on stiff COL1 probably reflect the highly-activated proliferation of cholangiocytes (ductular reaction) in liver fibrosis, and “atypical” proliferation of cholangiocytes commonly seen in patients with prolonged cholestatic liver diseases, such as primary sclerosing cholangitis or primary biliary cirrhosis [31, 32].

*C*. *sinensis* ESPs were the third component of the CCA microenvironment considered in this study, and ESP stimuli induced 3D morphological changes in biliary ductal plate. Some H69 cells in the 3D biliary ductal plate grown on a COL1 hydrogel interacted with it by sprouting; however, the majority maintained the layer structure during the entire experimental periods, independent of direct or gradient ESP application (Fig 4B and 4C). While no obvious changes in N-cadherin expression in H69 cells were observed, the expression of E-cadherin in H69 cells was increased gradually in a time-dependent manner during the experiments (Fig 7B), implying that the ESPs may cause H69 cells to exhibit more epithelial characteristics. However, we do not rule out the possibility that more intense stimulation, such as with a higher dose of ESPs and/or longer exposure times, could produce EMT-like effects in H69 cells.

We determined that ESPs are implicated in the acquisition of CCA malignant characteristics; increased invasion and migration. Single cell invasion by HuCCT1 cells was similarly increased by both direct and gradient ESP application, while migration increased significantly upon gradient application only. The concentration profile produced by computational simulation explained these differential effects on HuCCT1 cells; the average concentration of ESPs over the entire area of the 3D biliary ductal plate was estimated at over 1.5 μg/mL and the H69 cells forming the biliary ductal plate were exposed to high concentrations of ESPs (over 800 ng/mL), sufficient to induce significantly increased levels of IL-6 and TGF-β1 (Fig 4A, red dotted line). In contrast, the concentration of ESPs over the channel where HuCCT1 cells were seeded was considerably higher after direct application versus gradient application; yet, the magnitude of the effect on both migration and invasion was smaller (Fig 5). These results suggest multiple pathological effects of ESPs in the CCA microenvironment, such as the stimulation of normal tissues near the CCA and the chemoattraction of CCA cells.

It has been reported previously that *C*. *sinensis* ESP-triggered CCA cell migration/invasion is mediated by ERK1/2-NF-κB-MMP-9 and integrin β4-FAK/Src pathways, suggesting that ESPs may function as detrimental modulators of the aggressive progression of liver fluke-associated CCA [33, 34]. In the present study, the morphological features of HuCCT1 cells exposed to ESPs in 3D co-culture with H69 cells differed from those of HuCCT1 cells cultured alone. Co-cultured HuCCT1 cells exhibited increased motility, as represented by single cell invasion, while HuCCT1 cells exhibited aggregation in the 3D culture [14]. This implies that the interaction between HuCCT1 and H69 cells contributes to a change in HuCCT1 cell phenotype.

Cytokines generated by various types of cells within the tumor microenvironment play pro- or anti-tumorigenic roles, depending on the balance of different immune mediators and the stage of tumor development [35]. During liver fluke infection, chronically-inflamed epithelia are under constant stimulation to participate in the inflammatory response by continuous secretion of chemokines and cytokines. This creates a vulnerable microenvironment that may promote malignant transformation and even cholagiocarcinogenesis.

IL-6 is considered a proinflammatory cytokine that has typically pro-tumorigenic effects during infection. Liver cell lines, including H69 cells, preferentially take up *O*. *viverrini* ESPs by endocytosis, resulting in proliferation and increased secretion of IL-6 [7]. Elevated plasma concentrations of IL-6 are associated with a significant dose-dependent increase in the risk of opisthorchiasis-associated advanced periductal fibrosis and CCA [36]. The TGF-β-mediated signaling pathway is involved in all stages of liver disease progression from initial inflammation-related liver injury to cirrhosis and hepatocellular carcinoma [37]. A crude antigen from *C*. *sinensis* differentiates macrophage RAW cells into dendritic-like cells and upregulates ERK-dependent secretion of TGF-β, which modulates the host’s immune responses [38]. *C*. *sinensis* infection activates TGF-β1/Smad signaling promoting fibrosis in the livers of infected mice [19]. Additionally, it has been reported that the E/N-cadherin switch via TGF-β-induced EMT is correlated with cancer progression of CCA cells and the survival of patients with extrahepatic CCA [39, 40]. Consistent with these studies, we observed that the decreased E-cadherin and increased N-cadherin expression in ESP-exposed HuCCT1 cells (Fig 7A) was associated with increased secretion of IL-6 and TGF-β1 by H69 cells (Fig 6A) as well as by HuCCT1 cells, as reported previously [41]. The cytokine mediated-interaction between H69 and HuCCT1 cells was evaluated by means of siRNAs, which the levels of IL-6 and TGF-β1 secretion were suppressed in the culture supernatants of siRNA-IL-6 and -TGF-β1 H69 transfectants (Fig 6B). The suppression of these cytokines was correlated with an impairment of the change in E-/N-cadherin expression in HuCCT1 cells triggered by the ESPs (Fig 7C). This suggests that local accumulation of these cytokines, as the result of constitutive and dysregulated secretion of both cell types, promotes a more aggressive pathogenic process in the tumor milieu. Therefore, it is tempting to speculate that ESPs facilitate a positive feedback loop of elevated inflammatory cytokine secretion in both non-cancerous and cancerous cells, triggering an E/N-cadherin switch in HuCCT1 cells that subsequently increased invasion and/or migration mediated by the EMT. We will conduct future studies to explore this possibility.

In conclusion, HuCCT1 cells exhibited elevated single cell invasion after both direct and gradient ESP application, with increased migration occurring only after gradient treatment (ESPs applied to the basal side). These changes were caused by coordinated interactions between normal cholangiocytes, CCA cells and *C*. *sinensis* ESPs, which resulted in increased secretion of IL-6 and TGF-β1 and a cadherin switch in ESP-exposed cells. Therefore, the combined effects of these detrimental stimulations in both cancerous and non-cancerous bile duct epithelial cells during *C*. *sinensis* infection may facilitate a more aggressive phenotype of CCA cells, such as invasion/migration, resulting in more malignant characteristics of the CCA tumor. Our findings broaden our understanding of the molecular mechanism underlying the progression of CCA caused by liver fluke infection. These observations provide a new basis for the development of chemotherapeutic strategies to control liver fluke-associated CCA metastasis and thereby help to reduce its high mortality rate in the endemic areas.

## Materials and methods

### Culture medium and antibodies

Cell culture medium components were purchased from Life Technologies (Grand Island, NY), unless otherwise indicated. Polyclonal antibodies against the following proteins were purchased from the indicated sources: Ki-67 and integrin α6 (Abcam, Cambridge, UK); E-cadherin (BD Biosciences, San Jose, CA); N-cadherin (Santa Cruz Biotechnology, Santa Cruz, CA); glyceraldehyde-3-phosphate dehydrogenase (GAPDH; AbFrontier Co., Seoul, Korea). Horseradish peroxidase (HRP)-conjugated secondary antibodies were obtained from Jackson ImmunoResearch Laboratory (West Grove, PA). All other chemicals were obtained from Sigma-Aldrich (St. Louis, MO).

### Cell culture

Human HuCCT1 cholangiocarcinoma cells (originally established by Miyagiwa et al. in 1989 [42]) was maintained in RPMI 1640 medium supplemented with 1% (v/v) penicillin/streptomycin and 10% FBS. Human H69 cholangiocyte cells, which are SV40-transformed bile duct epithelial cells derived from non-cancerous human liver [43], were kindly provided by Dr. Dae Ghon Kim of the Department of Internal Medicine, Chonbuk National University Medical School, Jeonju, Korea. H69 cells were grown in DMEM/F12(3:1) containing 10% FBS, 100 U/mL penicillin, 100 μg/ml streptomycin, 5 μg/ml of insulin, 5 μg/ml of transferrin, 2.0 ×10^−9^ M triiodothyronine, 1.8 × 10^−4^ M adenine, 5.5 × 10^−6^ M epinephrine, 1.1 × 10^−6^ M hydrocortisone, and 1.6 × 10^−6^ M EGF. Both cell types were cultured at 37°C in a humidified atmosphere containing 5% CO_2_.

### Establishment of stably GFP expressing HuCCT1 cells

Clonal cell lines that stably expressed a green fluorescent protein (GFP) were generated by transfection of HuCCT1 cells. Briefly, HuCCT1 cells were grown to ~70% confluence and were transfected using Lipofectamine 2000 (Invitrogen, Calsbad, CA) and a pGFP-C1 vector (Clontech Laboratories, Inc., Palo Alto, CA) for 24 hours. To generate stable lines, the cells were cultured for 3 weeks in a complete medium containing 1 mg/ml G 418 disulfate salt (Sigma-Aldrich) that was changed every 2~3 days. Colonies with uniform GFP fluorescence were screened and two clonal cell lines with approximately similar levels of GFP overexpression were chosen for further experiments.

### Ethics statement

Adult *C*. *sinensis* specimens for the preparation of ESPs were obtained from infected, sacrificed New Zealand albino rabbits to collect adult worms. Animal care and experimental procedures were performed in strict accordance with the national guidelines outlined by the Korean Laboratory Animal Act (No. KCDC-122-14-2A) of the Korean Centers for Disease Control and Prevention (KCDC). The KCDC-Institutional Animal Care and Use Committee (KCDC-IACUC)/ethics committee reviewed and approved the ESPs preparation protocols (approval identification number; KCDC-003-11).

### Preparation of ESPs

The ESPs from *C*. *sinensis* adult worms were prepared as described previously [41]. Briefly, adult worms were recovered from the bile ducts of male New Zealand albino rabbits (12 weeks old) orally infected with ~500 metacercariae 12 weeks earlier. Worms were washed several times with cold phosphate-buffered saline (PBS) to remove any host contaminants. Five fresh worms were cultured in 1 mL of prewarmed PBS containing a mixture of antibiotics and protease inhibitors (Sigma-Aldrich) for 3 hours at 37 °C in a 5% CO_2_ environment. Then the culture fluid was pooled, centrifuged, concentrated with a Centriprep YM-10 (Merck Millipore, Billerica, MA) membrane concentrator, and filtered through a sterile 0.2-μm syringe membrane. After measuring the ESP protein concentration, the aliquots were stored at −80°C until use.

### Preparation of microfluidic device

The microfluidic device was prepared as described previously [14]. Briefly, the microfluidic devices were produced by curing polydimethylsiloxane (PDMS, Silgard 184, Dow Chemical, Midland, MI) overnight on a micro-structure-patterned wafer at 80°C. The device was punched to produce ports for the hydrogel and cell suspension injections. After sterilization, the device and s glass coverslip (24 × 24 mm; Paul Marienfeld, Germany) were permanently bonded to each other and the surfaces of microchannels in the device were coated with poly-D-lysine by treatment 1 mg/mL solution. The devices were stored under a sterile condition until use.

### 3D co-culture of H69 and HuCCT1 cells in a microfluidic device and ESPs treatment

The gel region of microfluidic device was filled with an unpolymerized COL1 solution (2.0 mg/mL, pH 7.4) and then placed in a 37°C humidified chamber to polymerize the hydrogel. EGF-depleted H69 medium containing 1% FBS was injected into the medium channels to prevent shrinkage of the COL1 hydrogel, and the devices were stored at 37°C in a 5% CO_2_ incubator until cell seeding. H69 cells (5 × 10^5^ cells) suspended in conditional medium (FBS-free, EGF-depleted) were loaded into one medium port. After filling a medium channel by the cells in the suspension by hydrostatic flow, the device was positioned vertically for 2 hours at 37°C in a 5% CO_2_ incubator to allow the cells to attach to the COL1 hydrogel wall by gravity. One day after seeding with H69 cells, HuCCT1-GFP cells suspended in conditional medium at 10 × 10^5^ cells/mL were seeded into the cell channel in a manner identical to the H69 cells. ESPs were diluted in conditional medium to a concentration of 4 μg/mL and then added either to the cell channel (direct application) or to the medium channel (gradient application). The medium was replaced every day with fresh conditional medium supplemented with ESPs (Fig 1B).

### Immunofluorescence analysis

H69 cells cultured in a microfluidic device were washed twice with PBS and fixed with a 4% paraformaldehyde solution for 30 minutes. A 0.1% Triton X-100 solution was treated to permeabilize the cell membranes for 10 minutes. The cells were incubated with 1% bovine serum albumin and primary antibodies against Ki67 or Integrin α6 (1:1000 dilution), followed by Alexa Fluor 488 secondary antibody (1:1000 dilution; Invitrogen). After staining with 4',6-Diamidino-2-Phenylindole (DAPI, 1:1000 dilution, Invitrogen), and rhodamine phalloidin (to stain F-actin, 1:200 dilution, Invitrogen), the cells were examined by a confocal laser-scanning microscope (LSM700; Carl Zeiss, Jena, Germany) and by fluorescent microscope (Axio Observer Z1; Carl Zeiss, Jena, Germany).

### Transfection with siRNA

We used the siRNAs (Ambion Silencer Select) of IL-6, TGF- β1, and scrambled oligonucleotide as a negative control from Thermo Fisher Scientific (Waltham, MA). H69 or HuCCT1 cells were seeded on 24-well culture plate and transiently transfected with either each or both target-specific siRNAs using Lipofectamine RNAiMAX (Invitrogen) according with the manufacturer’s protocols. Each siRNA transfection was performed in quadruplicate. After 24 hours, the transfection mixture on the cells was replaced with fresh culture medium. At 60 hour after transfection, H69 cells were depleted of FBS gradually, followed by incubation in conditional medium supplemented with 800 ng/mL ESPs for 48 hours. The culture supernatants from H69 cells were collected and clarified by brief centrifugation. Then, the 24 hour-ESP (800 ng/mL)-treated medium of HuCCT1 cells was replaced by these supernatants. The supernatants and cells were harvested after 48 hours and used for both ELISA and immunoblot analyses.

### Immunoblot analysis

HuCCT1 or H69 cells treated with 800 ng/mL ESPs, for the indicated times, were washed with ice-cold PBS and then lysed with a RIPA buffer containing a complete protease inhibitor cocktail (Sigma-Aldrich). Thirty μg of total soluble protein was separated by SDS-PAGE and electrophoretically transferred to a nitrocellulose membrane (Millipore, Bedford, MA). Membranes were probed with primary antibodies against E-cadherin (1:3000 dilution) or N-cadherin (1:1000). After incubation with host-specific secondary antibodies, the immunoreaction was detected with a West-Q-chemiluminescent substrate kit (GenDEPOT, Barker, TX) and quantitated by densitometric scanning of the X-ray film with a Fluor-S Multimager (Bio-rad, Hercules, CA). The blots were normalized for protein loading by washing in Blot-Fresh Western Blot Stripping Reagent (SignaGen Laboratories, Gaithersburg, MD) and re-probing with a polyclonal antibody against GAPDH (1:5000 dilution).

### Cytokine assay

Immunoreactive TGF-β1 and IL-6 in non-treated and ESP-treated (800 ng/mL) culture supernatants of H69 cells were quantitated in duplicate using commercially available ELISA kits (Enzo Life Sciences Inc., Ann Arbor, MI), according to the manufacturer’s instructions. The levels of each cytokine were determined by measuring absorbance at 450 nm and by comparing the absorbance values against a standard curve obtained using a four-parameter logistic curve fit.

### Computational simulation of ESP transport

COMSOL Multiphysics 5.0 (COMSOL, Sweden) software was used to simulate the concentration profile of ESPs. We assumed the molecular weight of ESPs to be 40 kDa, which is the molecular weight of cathepsin B, major component of ESPs [44]. The diffusion coefficients of ESPs in various milieus were estimated 8×10^−11^ m^2^/s in to be cell growth medium, 5.8×10^−11^ m^2^/s in a collagen hydrogel and 1.57×10^−12^ m^2^/s in a cell layer, based on previous studies on the VEGF transport [45], because the molecular weight of cathepsin B is similar to VEGF (42 kDa).

## References

[1] Fava G, Lorenzini I. Molecular pathogenesis of cholangiocarcinoma. Int J Hepatol 2012;2012:630543 10.1155/2012/630543 . 21994887 PMC3169310

[2] Tyson GL, El-Serag HB. Risk factors for cholangiocarcinoma. Hepatology. 2011;54:173–184. 10.1002/hep.24351 21488076 PMC3125451

[3] Rustagi T, Dasanu CA. Risk factors for gallbladder cancer and cholangiocarcinoma: similarities, differences and updates. J Gastrointest Cancer. 2012;43(2):137–47. 10.1007/s12029-011-9284-y 21597894

[4] Sripa B, Brindley PJ, Mulvenna J, Laha T, Smout MJ, Mairiang E, Bethony JM, et al The tumorigenic liver fluke Opisthorchis viverrini—multiple pathways to cancer. Trends Parasitol. 2012;28:395–407. 10.1016/j.pt.2012.07.006 . 22947297 PMC3682777

[5] IARC Working Group on the Evaluation of Carcinogenic Risks to Humans. Biological agents. Volume 100 B. A review of human carcinogens. IARC Monogr Eval Carcinog Risks Hum. 2012;100:1–441. 23189750 PMC4781184

[6] Kim T-S, Pak JH, Kim J-B, Bahk YY. Clonorchis sinensis, an oriental liver fluke, as a human biological agent of cholangiocarcinoma: a brief review. BMB Rep. 2016 2016/11//;49(11):590–7. 10.5483/BMBRep.2016.49.11.109 . 27418285 PMC5346318

[7] Chaiyadet S, Smout M, Johnson M, Whitchurch C, Turnbull L, Kaewkes S, Sotillo J, et al Excretory/secretory products of the carcinogenic liver fluke are endocytosed by human cholangiocytes and drive cell proliferation and IL6 production. Int J Parasitol. 2015;45:773–781. 10.1016/j.ijpara.2015.06.001 . 26187786 PMC4912216

[8] Ninlawan K, O’Hara SP, Splinter PL, Yongvanit P, Kaewkes S, Surapaitoon A, LaRusso NF, et al Opisthorchis viverrini excretory/secretory products induce toll-like receptor 4 upregulation and production of interleukin 6 and 8 in cholangiocyte. Parasitol Int. 2010;59:616–621. 10.1016/j.parint.2010.09.008 . 20887801 PMC3319364

[9] Pak JH, Kim IK, Kim SM, Maeng S, Song KJ, Na BK, Kim TS. Induction of cancer-related microRNA expression profiling using excretory-secretory products of Clonorchis sinensis. Parasitol Res. 2014;113:4447–4455. 10.1007/s00436-014-4127-y . 25217977

[10] Gkretsi V, Stylianopoulos T. Cell Adhesion and Matrix Stiffness: Coordinating Cancer Cell Invasion and Metastasis. Frontiers in oncology. 2018;8:145-. 10.3389/fonc.2018.00145 . 29780748 PMC5945811

[11] Brivio S, Cadamuro M, Strazzabosco M, Fabris L. Tumor reactive stroma in cholangiocarcinoma: The fuel behind cancer aggressiveness. World journal of hepatology. 2017;9(9):455–68. 10.4254/wjh.v9.i9.455 . 28396716 PMC5368623

[12] Maemura K, Natsugoe S, Takao S. Molecular mechanism of cholangiocarcinoma carcinogenesis. Journal of Hepato-Biliary-Pancreatic Sciences. 2014;21(10):754–60. 10.1002/jhbp.126 24895231

[13] Yamada D, Kobayashi S, Wada H, Kawamoto K, Marubashi S, Eguchi H, Ishii H, et al Role of crosstalk between interleukin-6 and transforming growth factor-beta 1 in epithelial-mesenchymal transition and chemoresistance in biliary tract cancer. Eur J Cancer. 2013;49:1725–1740. 10.1016/j.ejca.2012.12.002 . 23298711

[14] Won J, Ju J-W, Kim SM, Shin Y, Chung S, Pak JH. Clonorchis sinensis infestation promotes three-dimensional aggregation and invasion of cholangiocarcinoma cells. PLoS One. 2014;9:e110705 10.1371/journal.pone.0110705 25340585 PMC4207741

[15] Antoniou A, Raynaud P, Cordi S, Zong Y, Tronche F, Stanger BZ, Jacquemin P, et al Intrahepatic bile ducts develop according to a new mode of tubulogenesis regulated by the transcription factor SOX9. Gastroenterology. 2009;136:2325–2333. 10.1053/j.gastro.2009.02.051 . 19403103 PMC2743481

[16] Tanimizu N, Miyajima A, Mostov KE. Liver progenitor cells develop cholangiocyte-type epithelial polarity in three-dimensional culture. Mol Biol Cell. 2007;18:1472–1479. 10.1091/mbc.E06-09-0848 . 17314404 PMC1838984

[17] Roeder BA, Kokini K, Sturgis JE, Robinson JP, Voytik-Harbin SL. Tensile mechanical properties of three-dimensional type I collagen extracellular matrices with varied microstructure. J Biomech Eng. 2002;124:214 . 12002131 10.1115/1.1449904

[18] Maeng S, Lee HW, Bashir Q, Kim TI, Hong SJ, Lee TJ, Sohn WM, et al Oxidative stress-mediated mouse liver lesions caused by Clonorchis sinensis infection. Int J Parasitol. 2016;46:195–204. 10.1016/j.ijpara.2015.11.003 . 26718397

[19] Yan C, Wang L, Li B, Zhang B-B, Zhang B, Wang Y-H, Li X-Y, et al The expression dynamics of transforming growth factor-β/Smad signaling in the liver fibrosis experimentally caused by Clonorchis sinensis. Parasit Vectors. 2015;8:70 10.1186/s13071-015-0675-y . 25649869 PMC4329204

[20] De Assuncao TM, Sun Y, Jalan-Sakrikar N, Drinane MC, Huang BQ, Li Y, Davila JI, et al Development and characterization of human-induced pluripotent stem cell-derived cholangiocytes. Lab Invest. 2015;95:684–696. 10.1038/labinvest.2015.51 . 25867762 PMC4447567

[21] Ishida Y, Smith S, Wallace L, Sadamoto T, Okamoto M, Auth M, Strazzabosco M, et al Ductular morphogenesis and functional polarization of normal human biliary epithelial cells in three-dimensional culture. J Hepatol. 2001;35:2–9. . 11495037 10.1016/s0168-8278(01)00078-2

[22] Tanimizu N, Miyajima A, Mostov KE. Liver progenitor cells fold up a cell monolayer into a double-layered structure during tubular morphogenesis. Mol Biol Cell. 2009;20:2486–2494. 10.1091/mbc.E08-02-0177 . 19297530 PMC2675627

[23] Mizuguchi Y, Specht S, Isse K, Lunz JG, Demetris AJ: Biliary Epithelial Cells In: Monga SPS, ed. Molecular Pathology of Liver Diseases. Springer 2011; 27–51.

[24] Estrada MF, Rebelo SP, Davies EJ, Pinto MT, Pereira H, Santo VE, Smalley MJ, et al Modelling the tumour microenvironment in long-term microencapsulated 3D co-cultures recapitulates phenotypic features of disease progression. Biomaterials. 2016;78:50–61. 10.1016/j.biomaterials.2015.11.030 . 26650685

[25] Okabe H, Beppu T, Hayashi H, Horino K, Masuda T, Komori H, Ishikawa S, et al Hepatic stellate cells may relate to progression of intrahepatic cholangiocarcinoma. Ann Surg Oncol. 2009;16:2555 10.1245/s10434-009-0568-4 . 19548033

[26] Dianat N, Dubois-Pot-Schneider H, Steichen C, Desterke C, Leclerc P, Raveux A, Combettes L, et al Generation of functional cholangiocyte-like cells from human pluripotent stem cells and HepaRG cells. Hepatology. 2014;60:700–714. 10.1002/hep.27165 . 24715669 PMC4315871

[27] Huang P, Xu X, Wang L, Zhu B, Wang X, Xia J. The role of EGF-EGFR signalling pathway in hepatocellular carcinoma inflammatory microenvironment. J Cell Mol Med. 2014;18:218–230. 10.1111/jcmm.12153 . 24268047 PMC3930409

[28] Trussoni CE, Tabibian JH, Splinter PL, O’Hara SP. Lipopolysaccharide (LPS)-induced biliary epithelial cell NRas activation requires epidermal growth factor receptor (EGFR). PLoS One. 2015;10:e0125793 10.1371/journal.pone.0125793 . 25915403 PMC4411066

[29] Handorf AM, Zhou Y, Halanski MA, Li WJ. Tissue stiffness dictates development, homeostasis, and disease progression. Organogenesis. 2015;11:1–15. 10.1080/15476278.2015.1019687 . 25915734 PMC4594591

[30] Wells RG. The role of matrix stiffness in regulating cell behavior. Hepatology. 2008;47:1394–1400. 10.1002/hep.22193 . 18307210

[31] LaRusso NF, Wiesner RH, Ludwig J, MacCarty RL. Current concepts. Primary sclerosing cholangitis. N Engl J Med. 1984;310:899–903. 10.1056/NEJM198404053101407 . 6366557

[32] Roskams T, Desmet V. Ductular reaction and its diagnostic significance. Semin Diagn Pathol. 1998;15:259–269. . 9845427

[33] Pak JH, Bashir Q, Kim IK, Hong SJ, Maeng S, Bahk YY, Kim TS. Clonorchis sinensis excretory-secretory products promote the migration and invasion of cholangiocarcinoma cells by activating the integrin beta4-FAK/Src signaling pathway. Mol Biochem Parasitol. 2017;214:1–9. 10.1016/j.molbiopara.2017.03.002 . 28286026

[34] Pak JH, Shin J, Song IS, Shim S, Jang SW. Clonorchis sinensis excretory-secretory products regulate migration and invasion in cholangiocarcinoma cells via extracellular signal-regulated kinase 1/2/nuclear factor-kappaB-dependent matrix metalloproteinase-9 expression. Int J Parasitol. 2017;47:51–59. 10.1016/j.ijpara.2016.10.004 . 27919591

[35] Landskron G, De la Fuente M, Thuwajit P, Thuwajit C, Hermoso MA. Chronic inflammation and cytokines in the tumor microenvironment. J Immunol Res. 2014;2014:149185 10.1155/2014/149185 . 24901008 PMC4036716

[36] Sripa B, Thinkhamrop B, Mairiang E, Laha T, Kaewkes S, Sithithaworn P, Periago MV, et al Elevated Plasma IL-6 Associates with Increased Risk of Advanced Fibrosis and Cholangiocarcinoma in Individuals Infected by Opisthorchis viverrini. PLoS Negl Trop Dis. 2012;6:e1654 10.1371/journal.pntd.0001654 . 22629477 PMC3358341

[37] Dooley S, ten Dijke P. TGF-β in progression of liver disease. Cell Tissue Res. 2012;347:245–256. 10.1007/s00441-011-1246-y . 22006249 PMC3250614

[38] Wi HJ, Jin Y, Choi M-H, Hong S-T, Bae YM. Predominance of IL-10 and TGF-β production from the mouse macrophage cell line, RAW264.7, in response to crude antigens from Clonorchis sinensis. Cytokine. 2012;59:237–244. 10.1016/j.cyto.2012.04.021 . 22579699

[39] Araki K, Shimura T, Suzuki H, Tsutsumi S, Wada W, Yajima T, Kobayahi T, et al E/N-cadherin switch mediates cancer progression via TGF-beta-induced epithelial-to-mesenchymal transition in extrahepatic cholangiocarcinoma. Br J Cancer. 2011;105:1885–1893. 10.1038/bjc.2011.452 . 22068819 PMC3251878

[40] Duangkumpha K, Techasen A, Loilome W, Namwat N, Thanan R, Khuntikeo N, et al BMP-7 blocks the effects of TGF-β-induced EMT in cholangiocarcinoma. Tumor Biology. 2014 10 01;35(10):9667–76. 10.1007/s13277-014-2246-9 . 24969562

[41] Nam JH, Moon JH, Kim IK, Lee MR, Hong SJ, Ahn JH, Chung JW, et al Free radicals enzymatically triggered by Clonorchis sinensis excretory-secretory products cause NF-kappaB-mediated inflammation in human cholangiocarcinoma cells. Int J Parasitol. 2012;42:103–113. 10.1016/j.ijpara.2011.11.001 . 22138019

[42] Miyagiwa M, Ichida T, Tokiwa T, Sato J, Sasaki H. A new human cholangiocellular carcinoma cell line (HuCC-T1) producing carbohydrate antigen 19/9 in serum-free medium. In Vitro Cell Dev Biol. 1989;25:503–510. . 2544546 10.1007/BF02623562

[43] Grubman SA, Perrone RD, Lee DW, Murray SL, Rogers LC, Wolkoff LI, Mulberg AE, et al Regulation of intracellular pH by immortalized human intrahepatic biliary epithelial cell lines. Am J Physiol. 1994;266:G1060–G1070. 10.1152/ajpgi.1994.266.6.G1060 . 8023938

[44] Chen W, Wang X, Li X, Lv X, Zhou C, Deng C, Lei H, et al Molecular characterization of cathepsin B from Clonorchis sinensis excretory/secretory products and assessment of its potential for serodiagnosis of clonorchiasis. Parasit Vectors. 2011;4:149 10.1186/1756-3305-4-149 . 21794140 PMC3163202

[45] Jeong GS, Kwon GH, Kang AR, Jung BY, Park Y, Chung S, Lee S-H. Microfluidic assay of endothelial cell migration in 3D interpenetrating polymer semi-network HA-Collagen hydrogel. Biomed Microdevices. 2011;13:717–723. 10.1007/s10544-011-9541-7 . 21494794

